# Drug loss from Paclitaxel-Coated Balloons During Preparation, Insertion and Inflation for Angioplasty: A Laboratory Investigation

**DOI:** 10.1007/s00270-022-03164-5

**Published:** 2022-06-10

**Authors:** Bernd Faenger, Andreas Heinrich, Ingrid Hilger, Ulf Teichgräber

**Affiliations:** Deaprtement of Radiology, Jena University Hospital, Friedrich Schiller University Jena, Jena, Germany

**Keywords:** Drug-coated balloons, DCB, Catheter, Paclitaxel, Angioplasty, Peripheral artery disease

## Abstract

**Purpose:**

To investigate drug contamination of the working environment with paclitaxel drug-coated balloon (DCB) angioplasty due to loss of paclitaxel containing particles from the coating during DCB preparation, insertion, and inflation.

**Material and Methods:**

In an experimetal laboratory setting, drug loss during removal of the protective cover and insertion of the DCB through the hemostatic valve of the introducer sheath and after inflation was examined. In seven DCB types of different manufacturers, semi-quantitative image analysis was performed during five standardized tests cycles. Additionally, every DCB type passed one cycle of a wipe test and one cycle of air sampling.

**Results:**

By removing the protective cover, the paclitaxel-covered balloon surface was significantly reduced in 3 out of 7 products (*P* = 0.043). Overall, extend of decline ranged from 0.4 to 12%. In 6 of 7 products, powdered paclitaxel clusters dropped down upon removal of the protective cover (0.099 ng/cm^2^ up to approx. 22 ng/cm^2^). Contamination of the air was detected in none of the DCB types. When pushed through the vascular sheath, none of the investigated DCB types showed a significant loss of paclitaxel from the coated balloon surface. After balloon inflation, the paclitaxel-coated surface area varied between manufacturers ranging from 25.9 to 97.8%.

**Conclusion:**

In some DCB types, the removal of the protective cover already leads to a significant loss of paclitaxel and paclitaxel-coated surfaces. As a result, there will be a contamination of the workplace and a reduction in the therapeutic dose.

**Level of Evidence:**

No level of evidence.

**Supplementary Information:**

The online version contains supplementary material available at 10.1007/s00270-022-03164-5.

## Introduction

The use of paclitaxel drug-coated balloon (DCB) catheters to treat stenotic lesions in peripheral artery disease (PAD) is a routine procedure [1].

However, studies on efficacy of DCBs indicate that the success of the treatment differs across DCB types with different paclitaxel containing coating technologies [2, 3]. One possible cause may be that not with all products the theoretically assumed quantity of paclitaxel actually reaches the target lesion. There are several studies using blood vessel phantoms that have examined the loss of paclitaxel on its passage through the vessel [4–6]. In experimental models, it has been reported that at least 26 to 36% of the paclitaxel loaded on balloons with either urea matrix or iopromide coating is lost in the blood stream [7]. In a previous study, we used an experimental setup to investigate possible drug loss during vessel transfer. We focused on shear forces that may act on the coating and evaluated coating abrasion. Thereby, we found different susceptibility to abrasion across different DCB types [8].

However, to date, there are almost no studies that examine handling losses prior to the introduction of DCBs. One study sought to determine the coating durability of the Lutonix DCB (Becton, Dickinson, and Company, Franklin Lakes, New Jersey, USA) compared to the IN.PACT Admiral DCB (Medtronic, Dublin, Irland) and to evaluate the amount of paclitaxel that does not adhere to the balloon during simulated clinical procedural handling [9, 10]. Only few manufacturers have conducted studies to determine how much paclitaxel sticks to the valve [7]. Whether and how much of the drug is released from the balloon surface when the protective cover is removed has not yet been evaluated.

To clarify whether the handling of paclitaxel-coated DCBs may result in possible contamination of the work area with a possible hazard to medical personnel, the present investigation determines whether there is any loss of the paclitaxel-coated surface during the removal of the protective cover, during the insertion of the catheter through the hemostatic valve, and during balloon inflation.

## Materials and Methods

**Fig. 1:**
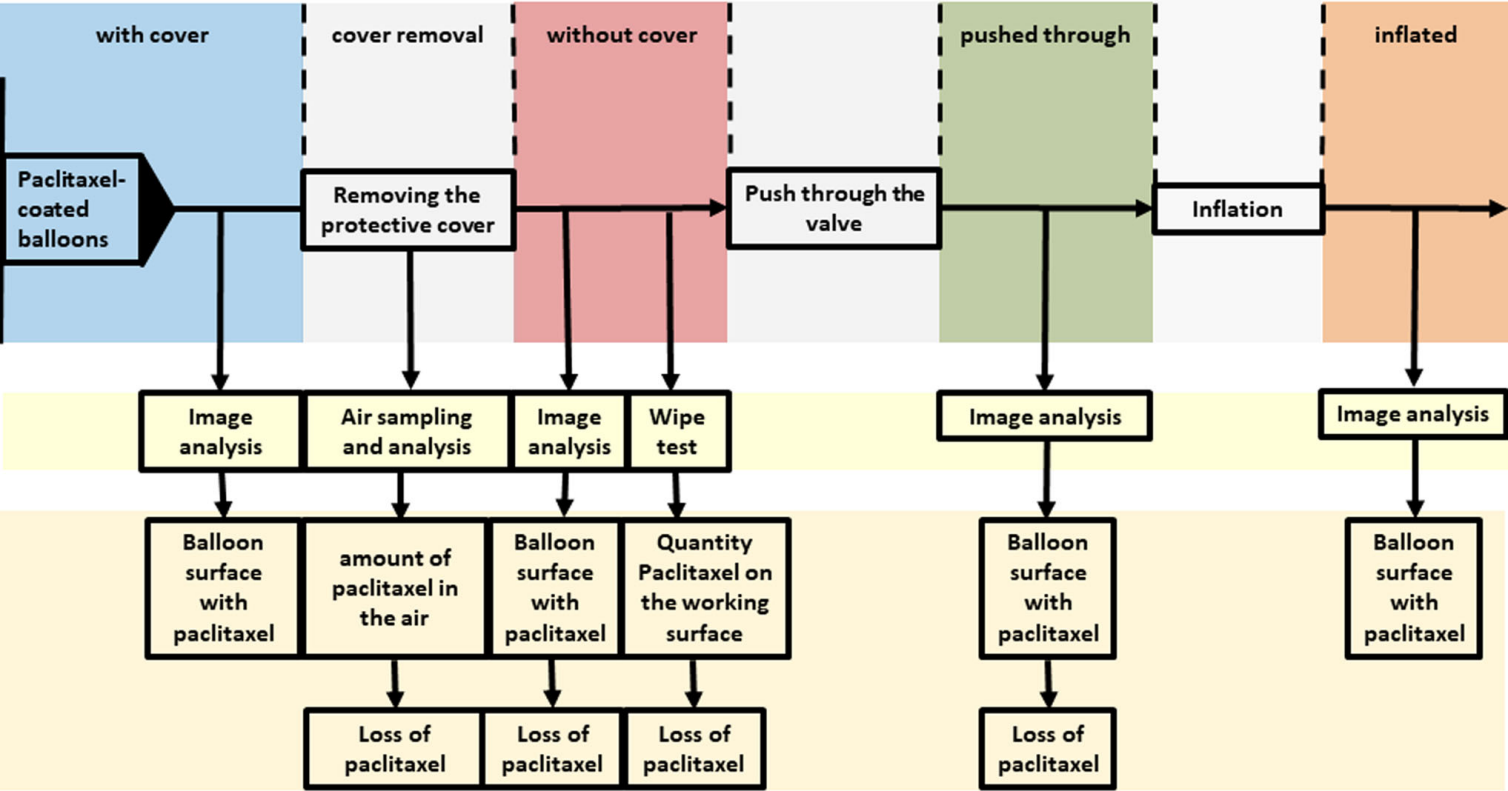
Study workflow diagram

The loss of drug coating from the DCBs was assessed using different methods at different time intervals during the preparation process of the DCB catheters (Fig. 1):

1. Identification of paclitaxel loss due to removal of the protective cover: The surface area of the DCB coating was determined semi-quantitatively before and after removal of the protective cover. For quantitative determination, the dropped coating was wiped off the working surface and measured. In addition, the amount of small paclitaxel particles, released into the air during removal of the protective cover, was measured.

2. Determination of paclitaxel loss caused by pushing the DCB through the hemostatic valve of the introducer sheath: The surface area of the DCB coating was determined semi-quantitatively before and after the balloon was pushed through the valve.

3. Measurement of the surface coated with paclitaxel after DCB inflation to obtain additional information regarding type and nature of the coating.

### Drug-Coated Balloon Catheters

**Fig. 2:**
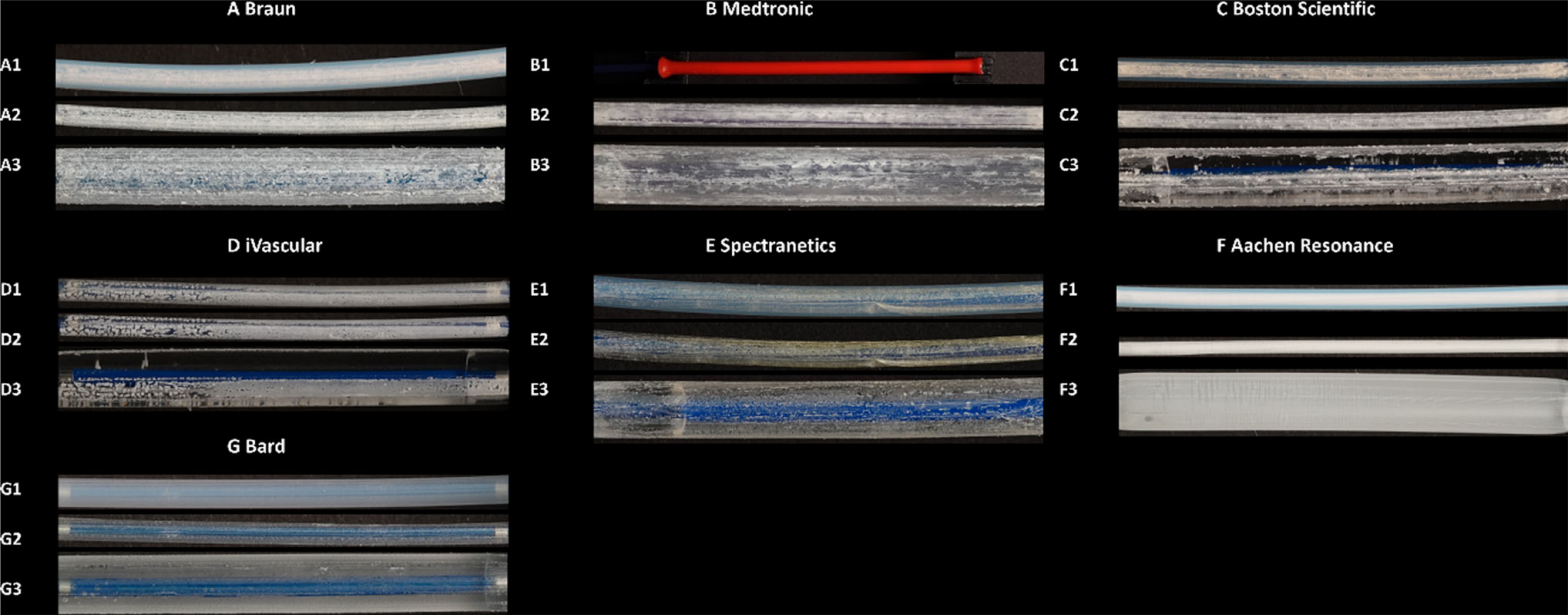
Samples of the DCBs of the seven manufacturers (A-G). With protective cover (A1-G1), without protective cover (A2-G2) and inflated (A3-G3)

The analysis included seven DCB types of equal size and length of seven manufacturers who used different paclitaxel dose and coatings (Table 1, Fig. 2). Overall, we evaluated a total of 49 DCB catheters. All products feature a balloon with a diameter of 5 mm and a length of 40 mm. The paclitaxel concentrations of DCBs ranged from 2 µg/mm^2^ to 3.5 µg/mm^2^. Depending on the manufacturer, the coatings of the products consist of the drug paclitaxel and one or more excipients to bind the drug to the balloon surface. The coated surfaces differ in structure (see comments Table 1, Fig. 2). The only non-transparent balloon cover is the one offered by the manufacturer Medtronic. Therefore, it was not possible to analyze the surface before removal of the protective cover.

**Table 1:** Manufacturer and product characteristics

Manufacturer	Product	Balloon length [mm]	Diameter [mm]	Paclitaxel Concentration [µg/mm^2^]	Additional Information Coating	Comment
Braun	SeQuent® Please OTW	40	5.0	3	The drug is embedded in a degradable delivery matrix (resveratrol)	The DCBs have a very thin, slightly powdery, structured coating
Medtronic	IN.PACT® Admiral®	40	5.0	3.5	Excipient: urea	Protective cover not transparent. The catheters have a plaque-like, scaly coating
Boston Scientific	Ranger™	40	5.0	2	Excipient: citrate Ester	The inflated balloons are not evenly coated on all sides; it is evident that they have been coated after folding. Their structure is powdery
iVascular	Luminor 35	40	5.0	3	Excipient: Organic ester. Lipophilic, biocompatible and biodegradable 20% Excipient, 80% Paclitaxel Catheter materials: Nylon/Pebax	The inflated balloons are not evenly coated on all sides; it is evident that they have been coated after folding. Their structure is powdery
Spectranetics	Stellarex™ 0.035 “ OTW	40	5.0	2	Hybrid paclitaxel (PTX) formulation: amorphous PTX + crystalline PTX + polythene glycol (PEG) excipient	The coating of the DCB is lacquer-like with uniform dot-like inclusions
Aachen Resonance	Elutax®-SV-OTW Fistula	40	5.0	2.2	Top layer 0.7 µg Dextran /mm^2^ as Excipient	The DCBs are clearly coated very thinly and very uniformly with a milky layer
Bard	Lutonix® 035	40	5.0	2	The drug coating is a non-polymer based formulation, consisting of pac. And polysorbate and sorbitol, with act as drug carrier	The coating is quite thin and lacquer-like, yet transparent

### Detection of Quantitative Drug Loss Using the Wipe Test

**Fig. 3:**
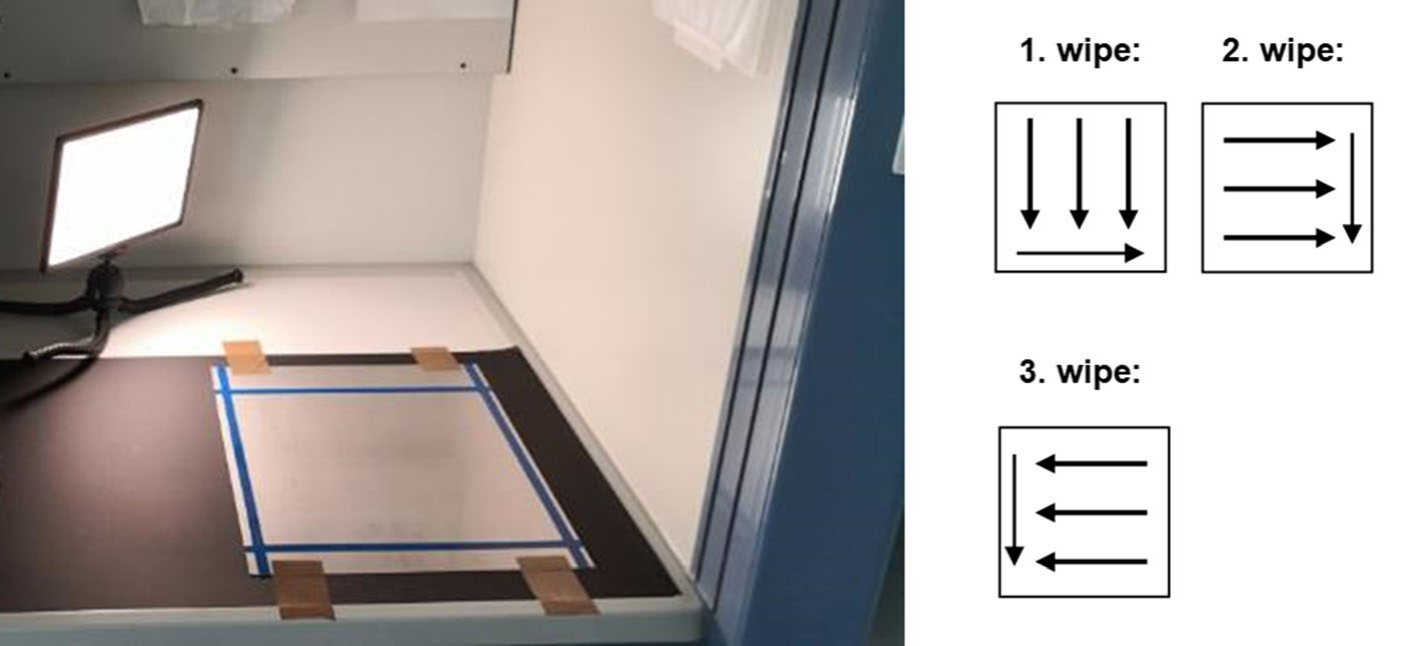
Wipe sampling test surface. A 30 cm x 30 cm steel plate and wiping direction

A wipe test (Fig. 3) was carried out to determine whether the work environment (surface/floor) was contaminated with paclitaxel when the protective covers were removed from the DCBs and to quantify the amount of paclitaxel dropped. One DCB (*n* = 1) from each manufacturer was used for the wipe test. The wipe sampling set employed was developed by the company BERNER International in cooperation with the Institute for Energy and Environmental Technology e.V. (IUTA) to detect contamination by CMR drugs (drugs with carcinogenic, mutagenic, and/or reproductive toxicity properties) at the workplace. The kit is designed for paclitaxel detection on smooth stainless steel surfaces. For this reason, a test area of 30 cm × 30 cm made of stainless steel was used for the experimental setup. The protective covers of the catheters were removed above the steel plate, and subsequently, the wipe test was performed according to the following instructions.

1. Three wipes were removed from the sample cup and placed on the lid of a polystyrene box.
2. Approximately 1 ml of the sampling solution was pipetted onto a cloth.
3. The test surface was wiped evenly in one direction with the moistened cloth, the cloth was folded once and wiped again once on the cleaning wipe front. The surface was wiped with one test cloth at a time in streaks in a single direction along the test surface.
4. The cloth was then returned to the sample cup.
5. The wiping procedure was repeated with the other two cloths as described under 1–4, alternating the wiping direction.
6. All cloths from one sample (one catheter) were returned to the respective labeled cup.
7. The gloves were changed after each sampling (after each catheter).

All samples were sent to the IUTA for external evaluation. The analyses were conducted according to SAA 2.2.2 (Determination of paclitaxel in wipe samples using LC–MS/MS, April 2019).

### Semi-Quantitative Image Analysis of DCB drug Loss from Removal of the Protective Cover and During Balloon Inflation

The focus of the present study was on the semi-quantitative evaluation of drug loss. To this end, five DCBs (*n* = 5) of each type were evaluated in a total of 35 test cycles. To guarantee standardized test conditions, the tests were carried out in self-developed 3D-printed test boxes made of PLA. These test boxes were adapted to the dimensions of the DCBs employed so that the handling of the DCBs could be accomplished with no additional vibrations. Vibrations might have resulted in an increased loss of paclitaxel. Also, these boxes provided optimal illumination of the balloons.

**Fig. 4:**
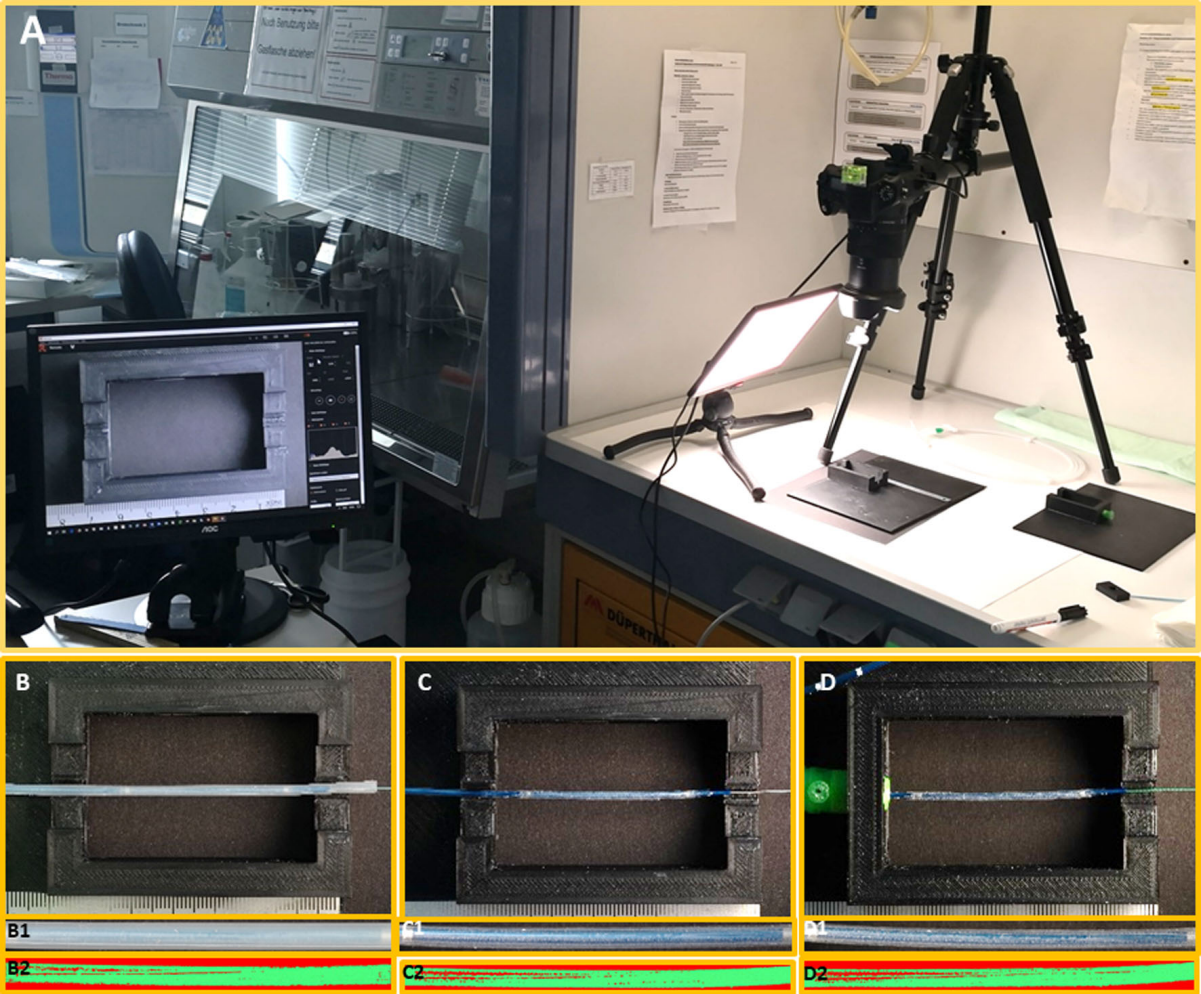
Exemplary test setup for the semi-quantitative evaluation of the paclitaxel-coated surface. A: Panoramic view of the test setup, B: Packed (with protective cover) exemplary DCB in the test box before removing the protective cover, B1: Camera photograph of the packed DCB to be evaluated, B2: Surface area of the coated balloon surface in the packed state, calculated by means of Trainable Weka Segmentation, C: Without cover (protective cover removed) exemplary DCB in the test box, C1: Camera photograph of the DCB without cover to be evaluated, C2: Surface area of the coated balloon surface without cover, calculated by means of Trainable Weka Segmentation, D: sample DCB without cover in the test box after pushing it through the valve, D1: Camera photograph for evaluation of the DCB after it was pushed through the valve, D2: Surface area of the coated balloon surface after the DCB was pushed through the valve, calculated by means of Trainable Weka Segmentation

Initially, the DCBs were placed in a first test box in a packed state (with protective cover) and photographed (Fig. 4, A, B, B1). In a second step, the protective covers were carefully removed from the DCBs still in the first test box and another photograph was taken without the protective cover (Fig. 4, C, C1). Unfolding was performed following a “material test” described by some manufacturers to show how firmly the paclitaxel adheres to the balloon [7, 10, 11]. The removal of the protective cover was captured at 250 fps with a high-speed camera (Online-Resource 1–3).

In a third step, the DCBs were pushed through the hemostatic valve into a second test box and then photographed in their pushed through state (Fig. 4, D, D1). The push-through was captured at 60 fps (Online-Resource 4–6). To document possible adhesions of abraded paclitaxel particles, all valves were photographed from the outside (SI. 1). In a fourth step, the catheters were inflated in the test box using a Medtronic inflator. The unfolding was recorded at 500 fps with a high-speed camera (Online-Resource 7–9).

Softwares ImageJ and Trainable Weka Segmentation [12] were used to analyze the dimensions of the paclitaxel-coated surfaces of the captured images/balloon surfaces of the DCBs (Fig. 4, B2-D2). Based on the dimensions of the paclitaxel-coated surfaces determined by Weka and the largest possible coated surface area (total surface area) at the defined times (with cover, without cover, pushed through, and inflated), the paclitaxel-coated surface proportions of the total surface were calculated in Excel. The entire visible surface of the balloons was used in the process. The photographs were taken in top view, thus 50% of the entire surface of the balloons was visible and considered. Only the relevant layer on the outer surface was evaluated in each case. In the folded state, the deeper layers were disregarded. Only surface portions completely without coating (“holes” in the coating) were evaluated as uncoated. For each image, the software was visually trained until only the surface portions with a coating were marked and “holes” in the coating were not marked. To reliably evaluate all image pixels, the visual evaluation was performed on a large high-resolution monitor. To guarantee a consistent evaluation, all images were analyzed by the same observer. Five identical products were used from each manufacturer (*n* = 5).

### Quantitative Detection of Paclitaxel Loss by Means of Air Samplings

**Fig. 5:**
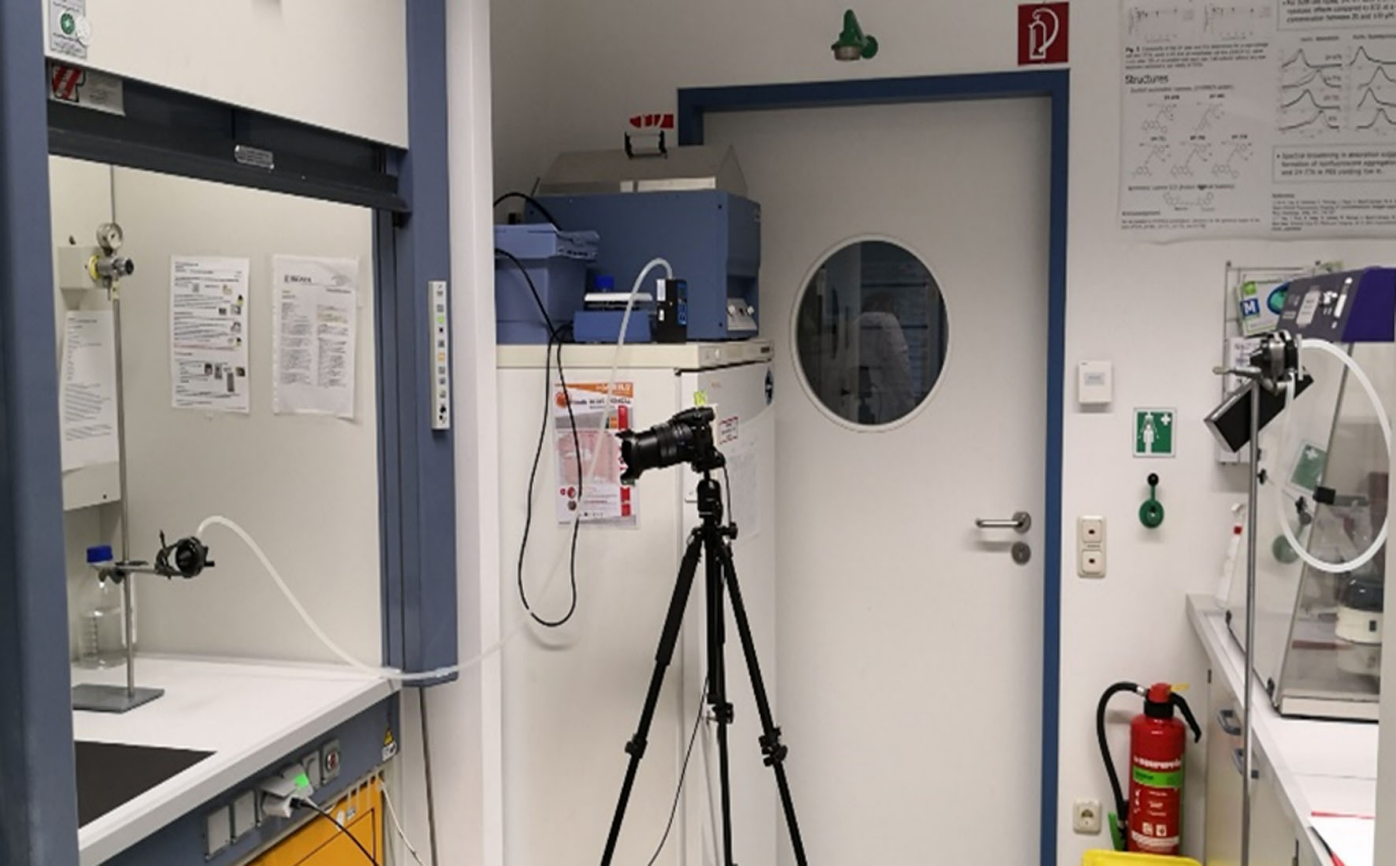
Experimental setup for the determination of paclitaxel exposure in the air when removing the protective cover

During the removal of the DCBs’ protective covers, air samples were taken by an external sampler and subsequently evaluated by IUTA (Institut für Energie- und Umwelttechnik e.V.). Only one DCB (*n* = 1) from each manufacturer (7 test cycles) was used for the air samples. In addition to the air sampling by means of a quartz filter, which was positioned at a distance of approx. 20 cm directly next to where the protective cover was removed, a reference sampling of the ambient air was performed at a distance of 2 m (Fig. 5).

### Statistics

Data were analyzed using SPSS Statistics 25 (IBM). A Friedman test was carried out, followed by the Wilcoxon test to compare paclitaxel-coated DCB surfaces before and after removal of the protective cover and after passage of the hemostatic valve. A p value < 0.05 was considered significant. Effect size was assessed and rated according to Cohen’s classification (*r* = 0.10 denotes a weak effect, *r* = 0.25 denotes a medium effect, *r* = 0.40 denotes a strong effect).

## Results

### Testing Results of the Balloon Surface with Protective Cover

#### Image Analysis

**Fig. 6:**
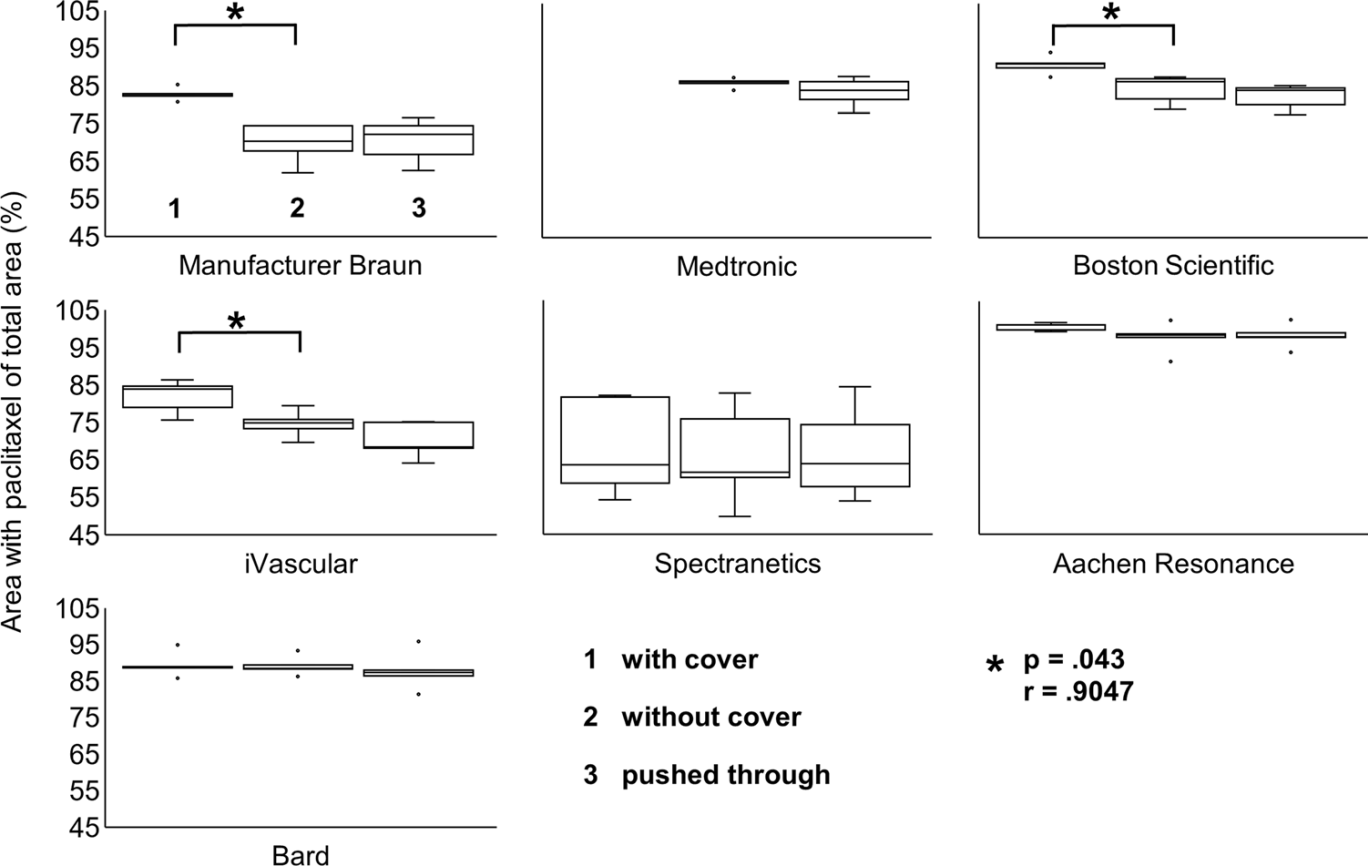
The removing of the protective cover resulted in a loss of the paclitaxel originally applied to the balloons. Semi-quantitative evaluation by Weka image analysis of 5 (*n* = 5) catheters per manufacturer. 100% is the maximum surface area that can be coated with paclitaxel. Coating of the catheters was evaluated in the packed state (1), after removing the protective cover (2) and after being pushed through the valve (3). Depicted are the percentage medians and the first and third quartiles. Minima and maxima are indicated by whiskers. The points plotted mark the outliers. The significant difference is represented according to Wilcoxon (*p* < 0.05). Cohen’s classification (1992) is used to assess the size of the effect: *r* = .10 denotes a weak effect, *r* = .25 denotes a medium effect, *r* = .40 denotes a strong effect

Regarding the balloon surface coated with paclitaxel, the product quality differed across manufacturers. With the protective cover still on, the median share of the balloon area coated with paclitaxel as a proportion of the total surface area was between 62.9% and 97.9% (Table 2, Fig. 6). For the Medtronic’s product, no data can be obtained in the packed state due to an optically impermeable protective cover.

**Table 2:** Results on quantitative drug loss (wipe test) and semi-quantitative drug loss

Manufacturer	Quantitative drug loss (wipe test)	Semi-quantitative drug loss				
	Paclitaxel concentration per area [ng/cm^2^]	Area of paclitaxel of total area (%)				
			with cover	without cover	pushed through	inflated
Braun	**22**^**^**^	Median	**82,4**	70,3*	72,2	71,4
		IQR	0,6	6,6	7,7	1,5
Medtronic	**12**^**^**^	Median	–	84,7	82,8	80,0
		IQR	–	0,5	4,6	3,9
Boston Scientific	**6,1**^**^**^	Median	**89,6**	85,0*	82,8	37,0
		IQR	1,2	5,2	4,3	3,5
iVascular	**3,3**^**^**^	Median	**83,9**	74,8*	68,3	25,9
		IQR	5,7	2,6	6,9	4,5
Spectranetics	**1,1**^**^**^	Median	62,9	60,9	63,1	55,9
		IQR	22,1	15,1	16,0	10,3
Aachen Resonance	0,099	Median	97,9	96,5	96,0	97,8
		IQR	1,2	0,9	1,2	6,5
Bard	< 0,009	Median	88,8	88,4	87,3	90,1
		IQR	0,3	1,0	1,5	3,2

Quantitative drug loss (wipe test): ^^^: Above reference value, the reference value is 0.1 ng/cm^2^; Semi-quantitative drug loss: IQR: Interquartile range; *: *p* = 0.043, *r* = 0.9047

### Testing Results of the Balloon Surface After Cover Removal

#### Air sampling and Analysis

No air contamination from paclitaxel-coated surfaces could be detected with removal of the protective DCB cover. All measured values were below the detection limit (less than 1 ng paclitaxel per sample) (Table 3).

**Table 3:** Paclitaxel detection in the room air after removal of the protective DCB cover

Manufacturer	Product	Paclitaxel [ng/sample]	Suction volume [L]	Paclitaxel [µg/m^3^]
Braun	SeQuent® Please OTW	< 1	16	< 0.06
Medtronic	IN.PACT® Admiral®	< 1	14	< 0.07
Boston Scientific	Ranger™	< 1	14	< 0.07
iVascular	Luminor 35	< 1	14	< 0.07
Spectranetics	Stellarex™ 0.035 “ OTW	< 1	14	< 0.07
Aachen Resonance	Elutax®-SV-OTW Fistula	< 1	14	< 0.07
Bard	Lutonix® 035	< 1	15	< 0.07
Background measurement		< 1	1.300	< 0.001

#### Image Analysis

Removing the protective cover from the DCBs led to loss of the paclitaxel-coated surface and thus to a contamination of the working surface. Overall, the semi-quantitative image analysis showed that loss of paclitaxel-coated surface area ranged from 0.4 to 12.1% (Table 2, Fig. 6). For the Medtronic’s product, the loss could not be assessed because no data can be obtained in the packed state due to an optically impermeable protective cover.

Regarding the balloon surface coated with paclitaxel, the product quality differed across manufacturers. Without cover, the median proportion of paclitaxel-coated areas of the total area of the balloon was between 60.9% and 96.5% (Table 2, Fig. 6). The proportion of the coated area in relation to the total area of the balloon in the state without cover also varied within the same DCB types (Fig. 6). In one of the DCB types, the proportion of the coated area ranged from 49.6 to 81.3% among the five measured DCBs. All the other DCB types showed a maximum difference of 12.6% within the same manufacturers.

#### Wipe Test

Removing the protective cover from the DCBs led to loss of the paclitaxel-coated surface and thus to a contamination of the working surface. In five DCB types, removal of the protective cover resulted in loss of paclitaxel with contamination of the test area above the reference value of 0.1 ng/cm^2^. In two DCB types, the amount of paclitaxel found on the test surface was below the reference value. The contamination of the test area ranged from 1.1 ng/cm^2^ to 22 ng/cm^2^ (Table 2).

### Testing Results of the Balloon Surface After Hemostatic Valve Passage

#### Image Analysis

Pushing the balloons through the hemostatic valve did not cause any measurable loss of paclitaxel-coated surface and therefore did not contaminate the work surface (p > 0.05 for all DCB types) (Table 2, Fig. 6). However, photographs of the valves show that coating particles adhered to the valve with all DCB types (**SI. 1**).

### Testing Results After Balloon Inflation

#### Image Analysis

**Fig. 7:**
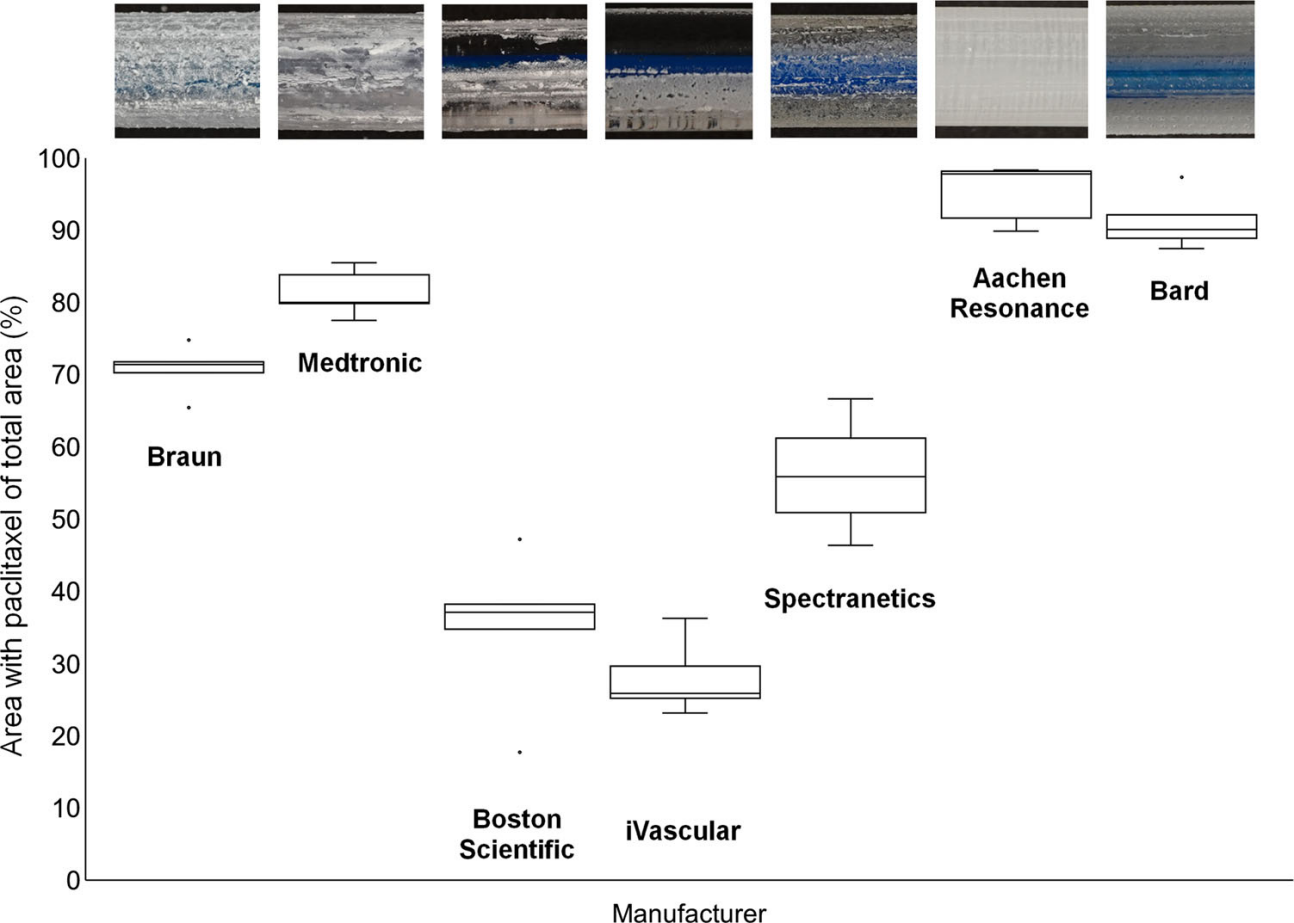
Percentage of the balloons’ surface area coated with paclitaxel in relation to the total area. Semi-quantitative analysis of five (*n* = 5) catheters per manufacturer. The coating of the catheters was evaluated in inflated state. 100% is the maximum surface area that could be coated with paclitaxel. Displayed are the percentage medians and the first and third quartiles for each product. The Whiskers indicate the minima and maxima. The points mark the outliers

The median of the paclitaxel-coated surface of the balloons varied between manufacturers ranging from 25.9 to 97.8% of the total surface (Table 2, Fig. 7). The products of the two manufacturers with the lowest proportion, 25.9% and 37%, of paclitaxel-coated surface area to total surface area have evidently not been coated over the total surface area depending on the different coating techniques. Excluding these two products, the proportion of paclitaxel-coated surface varies from 55.9 to 97.8%.

#### High-Speed Camera Recordings

High-speed camera recordings showed that with some manufacturers, particles burst off the surface explosively, whereas with other manufacturers, no detachment of particles can be visually detected (Online-Resource 7–9).

Recordings were obtained to visualize the loss of paclitaxel-coated surfaces during the above-described work steps, but not for measurement purposes (Online-Resource 1–9).

## Discussion

The wipe sampling revealed that the removal of the protective cover of six products used in the present study led to contamination of the work environment with paclitaxel. Five DCB catheter types were tested with a paclitaxel contamination above the reference value of 0.1 ng/cm^2^ [13, 14]. Air contamination was below the detection limit for all DCB types, and no significant loss of paclitaxel-coated surface was found after pushing the DCB through the introducer valve.

The research project MEWIP (monitoring effect study for wipe samples in pharmacies) was carried out by the German employers’ liability insurance association for health service and welfare work (BGW). The primary task of the BGW is to inform employees about occupational and commuting accidents, occupational diseases, and work-related health hazards, and to compensate them in the event of an emergency. In the scope of the MEWIP project, quarterly wipe samples were taken in 130 hospital and office pharmacies over a period of two years (2006–2007) to assess workplace exposure to eight different cytostatic drugs. Based on the 90th percentile of the contamination values, a performance-based guidance value of 0.1 ng /cm^2^ had been derived. However, the MEWIP reference value does not provide information about health risks for medical personnel and does not represent a permissible exposure limit. It rather can be taken as a reference point in the context of risk assessment. For higher concentrations, the work process should be carefully reassessed with regard to the release of cytostatic drugs and possible deficiencies or contamination routes (e.g., through leaks or malfunctions of the ventilation system) should be fixed [14].

In health care settings, dermal uptake is considered the most likely route of occupational exposure to most hazardous drugs, especially low-molecular-weight antineoplastic drugs [15]. Inhalation of aerosolized droplets or vapors, accidental hand-to-mouth ingestion following contact with contaminated surfaces, and needles or other sharps are also possible routes of exposure. In some cases, skin absorption may be a more important route of exposure than inhalation, especially for non-volatile hazardous chemicals which remain on work surfaces for long periods of time and may not be noticed by the employees. Antineoplastics enter the body through the respiratory tract and skin if protective measures are not properly applied. In the operating room, drug residues can accumulate due to the often-high number of daily patients. Unintentional carry-over can easily occur through contaminated gloves, protective clothing, footwear, wheels of transport trolleys, etc. In addition, swirling up of the accumulated residues by moving personnel is conceivable, so that contamination of the air can occur over time.

European and national regulations stipulate the identification of the exposure of workers with CMR substances (those with carcinogenic, mutagenic, and/ or reproductive toxicity properties) and the regular check of the effectiveness of protective measures taken by the employer (Council Directive 98/24/EC, 1998; Council Directive 2004/37/EC, 2004; German Ordinance on Hazardous Substances, 2010). However, to date, no threshold limit value has been established for paclitaxel. Thus, care should be taken when preparing the DCBs to avoid contaminating the workspace. A simple measure would be to uncover the DCBs over a defined and easily cleanable surface.

In our study, we could not measure any air contamination with paclitaxel. However, the extent to which air contamination may occur under working conditions in the angio suite remains unclear. Different rooms can have different air flows. However, in the experiment, the distance between the DCB and the air filter was chosen to be quite small at 20 cm. Therefore, it can be assumed that a low contamination with very small particles in the air would have been detected. It can be assumed that in clinical practice, there is less risk to the personnel from air contamination.

In our study, we found drug-loss from DCBs before passage through the arteries toward the target lesion. Drug loss differed across the assessed DCB types. Pictures of the inflated balloons show that two manufacturers are not coating their products evenly. Only those areas are coated which are located on the outside when the balloons are not inflated. Besides, there are considerable differences in the type of coating, ranging from rather powdery or flaky to lacquer-like. The results further illustrate that not all manufacturers offer the same level of product quality in terms of size of the coated surface area. In the five catheters from one manufacture, for example, the dimension of the coated area varies by up to 31.7% of the total balloon surface (without cover). Thus, it can be assumed that results of treatment may vary depending on the manufacturer, but also on the fact whether a manufacturer guarantees a constant quality of a product.

Whether shortfall of paclitaxel, early mechanical damaging of the DCB surface, as well as differences across DCB types in this respect does affect effectiveness and safety of the treatment remains uncertain [16]. At worst, detached larger particles could be swept along by the bloodstream too early, i.e., before the balloon surface of the DCB reaches the target vessel wall, and, as a result of an accumulation of the swept-along particles, could trigger thromboses at other, already constricted sections in the vascular system [17–19]. On the other hand, an over-stable coating might reduce the treatment success since not enough paclitaxel is released into the arterial wall [20]. However, paclitaxel dose at the target lesion also depends on the atherosclerotic condition of the artery itself [21]. Overall, the varying dimensions of the surfaces coated with paclitaxel can partly explain the fact that the effectiveness of treatment depends on the product used [2, 3, 22].

## Limitations

For both the wipe test and air samplings, only one DCB per manufacturer was used. Thus, we could not consider variability in quantitative measurement results within DCB types. In terms of handling, the wipe test in the present study was performed under conditions closer to practice than the semi-quantitative analysis test. The DCB was withdrawn quickly and “freely held,” as is usual in clinical routine. However, the semi-quantitative image analysis under precisely defined repeatable conditions with five products from each manufacturer showed comparable results to the wipe test. In addition, the extent to which air contamination occurs under working conditions in the clinic probably differs from the experimental setting. In our experiment, the distance between the DCB and the air filter was chosen to be quite small to detect very small particle amounts. Risk of harm from air contamination in the clinical practice with greater distances is probably low. A point of criticism about the semi-quantitative image analysis could be that only half of the total DCB area was assessed because the photographs were taken from above. However, the size of the area considered for the analysis can be regarded as sufficiently representative. Furthermore, in one of the DCB types, it was not possible to analyze the surface with the protective cover because a non-transparent balloon cover. Since this is the only missing value, it does not bias the overall study. The study did not investigate whether the amounts of paclitaxel on the DCBs reported by the manufacturers were correct. Since all manufacturers claim to coat the balloons uniformly, it can be assumed that a loss of coating also means a corresponding loss of paclitaxel. Although the wipe test and air analysis determined the amount of paclitaxel loss, they do not allow any conclusions on the amount of paclitaxel remaining on the balloon. Finally, as investigators of our study were not blinded, bias cannot be completely excluded.

## Conclusions

In some of the assessed DCBs, we found that removal of the protective cover led to a significant loss of the paclitaxel-coated surface. As a result, contamination of the workplace with paclitaxel might lead to adverse health effects. Therefore, as long as there are no concrete recommendations for the handling of paclitaxel, care should be taken when preparing DCBs. In addition, less paclitaxel can reach the target lesion, which may compromise the effectiveness of the treatment. The proportion of the total surface area coated with paclitaxel after balloon inflation differs considerably between manufacturers. In the future, a uniform approval procedure to verify the general stability of balloon coatings should be established for the regulatory approval of DCBs.

## Supplementary Information

Below is the link to the electronic supplementary material.

Supplementary file1 (MP4 8883 KB)

Supplementary file2 (MP4 4300 KB)

Supplementary file3 (MP4 6628 KB)

Supplementary file4 (MP4 3222 KB)

Supplementary file5 (MP4 2731 KB)

Supplementary file6 (MP4 24207 KB)

Supplementary file7 (MP4 19980 KB)

Supplementary file8 (MP4 17504 KB)

Supplementary file9 (MP4 17504 KB)

Supplementary file10 (DOCX 678 KB)

## References

[1] Teichgräber U (2021). Paclitaxel-coated balloon angioplasty in the real world: jack-of-all-trades?. Cardiovasc Intervent Radiol.

[2] Klumb C, Lehmann T, Aschenbach R, Eckardt N, Teichgraber U (2019). Benefit and risk from paclitaxel-coated balloon angioplasty for the treatment of femoropopliteal artery disease: a systematic review and meta-analysis of randomised controlled trials. EClinicalMedicine.

[3] Katsanos K, Kitrou P, Spiliopoulos S, Diamantopoulos A, Karnabatidis D (2016). Comparative effectiveness of plain balloon angioplasty, bare metal stents, drug-coated balloons, and drug-eluting stents for the treatment of infrapopliteal artery disease: systematic review and bayesian network meta-analysis of randomized controlled trials. J Endovasc Ther.

[4] Kaule S, Minrath I, Stein F (2015). Correlating coating characteristics with the performance of drug-coated balloons—a comparative in vitro investigation of own established hydrogel- and ionic liquid-based coating matrices. PLoS ONE.

[5] Seidlitz A, Kotzan N, Nagel S (2013). In vitro determination of drug transfer from drug-coated balloons. PLoS ONE.

[6] Kempin W, Kaule S, Reske T (2015). In vitro evaluation of paclitaxel coatings for delivery via drug-coated balloons. Eur J Pharm Biopharm.

[7] Kelsch B, Scheller B, Biedermann M (2011). Dose response to Paclitaxel-coated balloon catheters in the porcine coronary overstretch and stent implantation model. Invest Radiol.

[8] Heinrich A, Engler MS, Güttler FV, Matthäus C, Popp J, Teichgräber UKM (2020). Systematic evaluation of particle loss during handling in the percutaneous transluminal angioplasty for eight different drug-coated balloons. Sci Rep.

[9] Ward C, Mena CI (2014). TCT-289 detection of paclitaxel contamination resulting from the simulated clinical use of drug coated balloon catheters. J Am Coll Cardiol.

[10] Schorn I, Malinoff H, Anderson S (2017). The Lutonix® drug-coated balloon: a novel drug delivery technology for the treatment of vascular disease. Adv Drug Deliv Rev.

[11] Lutonix 035, Drug Coated Balloon, PTA Catheter. C. R. Bard, Inc. . 2017. http://www.lutonixdcb.com/pad-treatment.php. Accessed 21.10.2021 2021

[12] Arganda-Carreras I, Kaynig V, Rueden C (2017). Trainable Weka Segmentation: a machine learning tool for microscopy pixel classification. Bioinformatics.

[13] Kiffmeyer TK, Tuerk J, Hahn M (2013). Application and assessment of a regular environmental monitoring of the antineoplastic drug contamination level in pharmacies—the MEWIP project. Ann Occup Hyg.

[14] Wohlfahrtspflege BfGu. Zytostatika im Gesundheitsdienst. Berufsgenossenschaft für Gesundheitsdienst und Wohlfahrtspflege (BGW); Erstveröffentlichung 04/2008, Stand 02/2019

[15] Connor TH, Smith JP (2016). New approaches to wipe sampling methods for antineoplastic and other hazardous drugs in healthcare settings. Pharm Technol Hosp Pharm.

[16] Abadal JM, Vazquez E, Morales M, Toro A, Quintana M, Araujo M (2016). Pharmacokinetic evaluation of two paclitaxel-coated balloons with different drug load in a short-term porcine study. Cardiovasc Intervent Radiol.

[17] Katsanos K, Spiliopoulos S, Kitrou P, Krokidis M, Paraskevopoulos I, Karnabatidis D (2020). Risk of death and amputation with use of paclitaxel-coated balloons in the infrapopliteal arteries for treatment of critical limb ischemia: a systematic review and meta-analysis of randomized controlled trials. J Vasc Interv Radiol.

[18] Dan K, Shlofmitz E, Khalid N (2020). Paclitaxel-related balloons and stents for the treatment of peripheral artery disease: Insights from the Food and Drug Administration 2019 Circulatory System Devices Panel Meeting on late mortality. Am Heart J.

[19] Katsanos K, Spiliopoulos S, Kitrou P, Krokidis M, Karnabatidis D (2018). Risk of death following application of paclitaxel-coated balloons and stents in the femoropopliteal artery of the leg: a systematic review and meta-analysis of randomized controlled trials. J Am Heart Assoc.

[20] Granada JF, Stenoien M, Buszman PP (2014). Mechanisms of tissue uptake and retention of paclitaxel-coated balloons: impact on neointimal proliferation and healing. Open Heart.

[21] Fernández-Parra R, Laborda A, Lahuerta C (2015). Pharmacokinetic study of paclitaxel concentration after drug-eluting balloon angioplasty in the iliac artery of healthy and atherosclerotic rabbit models. J Vasc Interv Radiol.

[22] Teichgräber UK, Klumb C (2017). Drug-coated balloon angioplasty in femoropopliteal arteries—is there a class effect?. Zentralbl Chir.

