# Correction to: Drug Loss from Paclitaxel Coated Balloons During Preparation, Insertion and Inflation for Angioplasty: A Laboratory Investigation

**DOI:** 10.1007/s00270-022-03260-6

**Published:** 2022-08-31

**Authors:** Bernd Faenger, Andreas Heinrich, Ingrid Hilger, Ulf Teichgräber

**Affiliations:** Department of Radiology, Jena University Hospital, Friedrich Schiller University Jena, Jena, Germany

## Correction to: Cardiovasc Intervent Radiol (2022) 45:1186–1197 https://doi.org/10.1007/s00270-022-03164-5

In the original publication, one supplementary file is missing. Consequentially, supplementary files 5, 6, 7 and 8 are wrongly named and supplementary files 8 and 9 are duplicates. Here are all supplementary files with correct names and in correct order.

## Electronic supplementary material

Below is the link to the electronic supplementary material.

Supplementary material 1 (MP4 8883 kb)

Supplementary material 2 (MP4 4301 kb)

Supplementary material 3 (MP4 6629 kb)

Supplementary material 4 (MP4 3222 kb)

Supplementary material 5 (MP4 2739 kb)

Supplementary material 6 (MP4 2731 kb)

Supplementary material 7 (MP4 24207 kb)

Supplementary material 8 (MP4 19981 kb)

Supplementary material 9 (MP4 17504 kb)

