# Supplementary material for: Drug loss from Paclitaxel-Coated Balloons During Preparation, Insertion and Inflation for Angioplasty: A Laboratory Investigation
Source: Cardiovasc Intervent Radiol. 2022 Jun 10;45(8):1186–97. doi: 10.1007/s00270-022-03164-5 (PMC9307540; doi:10.1007/s00270-022-03164-5)
Supplement: Supplementary file 10 — Supplementary file10 (DOCX 678 KB) [file 270_2022_3164_MOESM10_ESM.docx]

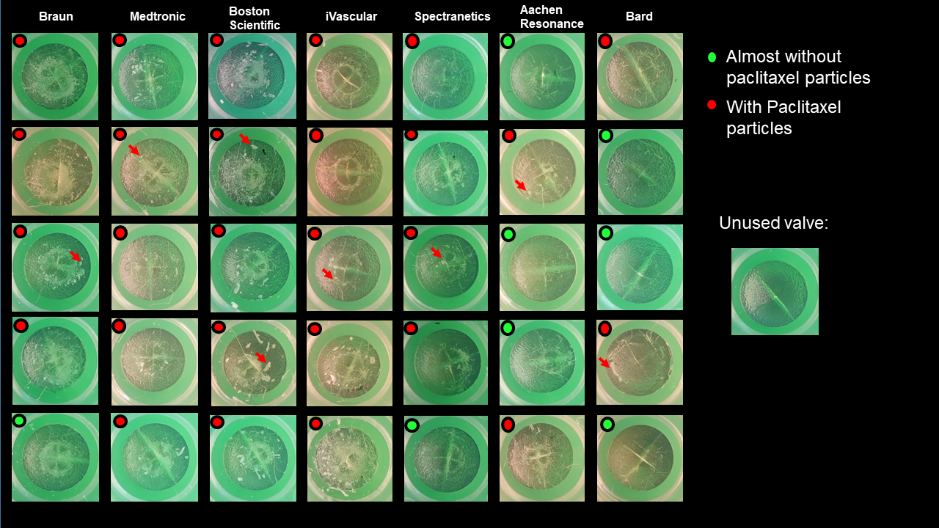


**SI. 1 Only few valves show almost no particles on their outside after the balloons have been pushed through.** Red arrow: Example particles
